# Some Notes Concerning Preparation of Teeth for Microscopic Study

**Published:** 1902-08

**Authors:** Martha Anderson


### SOME NOTES CONCERNING PREPARATION OF TEETH FOR MICROSCOPIC STUDY.[1]

BY MARTHA ANDERSON, M.D.

The results obtained in working teeth for the past few years do not justify a paper on the subject, but these few notes are given to tabulate results,—successes and failures,—hoping to bring out criticisms on the methods used.

In decalcification the results have been far from satisfactory. In order to protect the dentine and get the pulp and dentine *in situ,* the slower methods of decalcification are better, but the delay has disadvantages. The acid mixtures penetrate and destroy the staining properties of the tissues and cause distortion. In order to save the pulp the apical foramina were sealed with collodion or sealing-wax; this has not been satisfactory, as the sealing material comes off in spite of careful handling.

Nitric acid in weak solution gives slow decalcification. A two per cent. solution, with frequent changing every few days, took over one month to give results. Lee says nitric acid causes no swelling and does not injuriously attack tissue elements. This has not been my experience, as it has caused much distortion and the tissue elements have been largely destroyed. It does, however, give fair sections of dentine.

---

[1] Read before the Section on Stomatology, American Medical Association, Saratoga Springs, June, 1902.

Kleinenberg's method (picric acid, 100 parts; sulphuric acid, 2 parts; water, 300 parts) is exceedingly slow, requiring several (five) months.

Schaeffer's recipe preserves the dentine, but the tissue elements are distorted and staining properties destroyed.

Von Ebner's solution took four months; dentine preserved; tissue elements distorted and stained badly, though not so badly as the others.

Chromic acid (Boll) gradually preserves dentine (two months); tissues distorted.

Haug's method (phloroglucin, 1; nitric acid, 10; water, 100) is rapid and has the advantage of penetrating dentine; the pulp tissue, however, is distorted and stains badly.

Huber's recipe (nitric acid, 5 parts; hydrochoric acid, ½; water, 100) is rapid, requiring only a few days; it also causes distortion.

H. Smith's solution (hydrochloric acid, ten per cent., 12 c.c., sixteen hours, and nitric acid, 1.5 c.c., forty-six hours; nitric acid, 1.5 c.c., sixty-six hours) acts rapidly, entirely destroying the dentine and pulp-structure.

Chromic acid, five per cent (H. Smith) is rapid, eight to nine days, and destroys dentine and structure of pulp.

Andrew's solution (chromic acid, 140; nitric acid, 6; water, 400) is the only one that has given me any results that I can call good. Its disadvantages are its very rapid action; the solution in full strength completely destroying the entire tooth in a few hours. I have gotten results from using half strength and changing very frequently about every hour. If the tooth is forgotten for a few hours, it is apt to be destroyed. If one is required to give attention to other matters for a time, the specimen must be removed from the solution and placed in water, and returned to the solution again when convenient to watch it. Even then decalcification is apt to take place unevenly, and sometimes in spite of great care the pulp is attacked and partly destroyed by the acid. After this solution, sections have stained very well.

In preparing pulps for microscopic study, they were removed from fresh teeth and hardened in Müller's fluid. Flemming or formalin have given the best results. For staining cellular elements a good hæmatoxylin combined with eosin or Van Giesen's method is very satisfactory.

As a special nerve-stain, Weigert's method for medullated nerves is as follows: Harden in Müller's fluid; stain in a saturated solution of neutral copper acetate twenty-four hours; wash and pass into a stain composed of hæmatoxylin, 1; alcohol, 10; water, 90; saturated solution lithium carbonate, 1, for twenty-four hours. Then rinse in water and decolorize in borax, 2; potassium ferrocyanide, 2.5; water, 200. The nerve will stand out a deep brown compared to the other tissues. In studying sections by this process the nerve-trunks can be seen running from the apex of the pulp and branching off higher up, the fibrils running along the outer edge of the pulp just *beneath* the layer of odontoblasts. In a few cases I have been able to trace the nerve-fibrils out between the odontoblasts, but not into the dentine.

In pulps hardened in Hermann's recipe the nerves can also be traced. In pulps prepared by Weigert's recipe the pulp stones (?) are stained dark brown as the nerves. If they are pulp-stones, why do they stain thus?

So far the stains used for fibrous tissues and fat have been unsuccessful, but I have not yet completed my work in this line.

